# Characteristics and Long-Term Ablation Outcomes of Supraventricular Arrhythmias in Hypertrophic Cardiomyopathy: A 10-Year, Single-Center Experience

**DOI:** 10.3389/fcvm.2021.766571

**Published:** 2021-11-12

**Authors:** Hong-Da Zhang, Lei Ding, Si-Xian Weng, Bin Zhou, Xiao-Tong Ding, Li-Xing Hu, Ying-Jie Qi, Feng-Yuan Yu, Tian-Jie Feng, Jing-Tao Zhang, Pi-Hua Fang, Wei Hua, Shu Zhang, Min Tang

**Affiliations:** Arrhythmia Center, State Key Laboratory of Cardiovascular Disease, Fuwai Hospital, National Center for Cardiovascular Diseases, Chinese Academy of Medical Sciences and Peking Union Medical College, Beijing, China

**Keywords:** hypertrophic cardiomyopathy, supraventricular arrhythmia, atrial fibrillation, catheter ablation, outcomes

## Abstract

**Background:** A variety of supraventricular arrhythmias (SVAs) may occur in patients with hypertrophic cardiomyopathy (HCM). The characteristics and long-term ablation outcomes of different types of SVAs in HCM have not been comprehensively investigated.

**Methods:** We retrospectively enrolled 101 consecutive patients with HCM who were referred to the electrophysiology and arrhythmia service from May 2010 to October 2020. The clinical features and ablation outcomes were analyzed.

**Results:** Seventy-eight patients had SVAs, which comprised 50 (64.1%) cases of atrial fibrillation (AF), 16 (20.5%) of atrial flutter (AFL), 15 (19.2%) of atrioventricular reentrant tachycardia (AVRT), 11 (14.1%) of atrial arrhythmia (AT), and 3 (3.8%) of atrioventricular nodal reentrant tachycardia (AVNRT). Thirty-four patients underwent catheter ablation and were followed up for a median (interquartile range) of 58.5 (82.9) months. There was no recurrence in patients with non-AF SVAs. In patients with AF, the 1- and 7-year AF-free survival rates were 87.5 and 49.5%, respectively. A receiver operator characteristic analysis showed that a greater left ventricular end-diastolic dimension (LVEDD) was associated with a higher recurrence of AF, with an optimum cutoff value of 47 mm (c-statistic = 0.91, *p* = 0.011, sensitivity = 1.00, specificity = 0.82). In Kaplan–Meier analysis, patients with a LVEDD ≥ 47 mm had worse AF-free survival than those with a LVEDD <47 mm (log-rank *p* = 0.014).

**Conclusions:** In this unique population of HCM, AF was the most common SVA, followed in order by AFL, AVRT, AT, and AVNRT. The long-term catheter ablation outcome for non-AF SVAs in HCM is satisfactory. A greater LVEDD predicts AF recurrence after catheter ablation in patients with HCM.

## Introduction

Tachyarrhythmias often cause palpitations and can precipitate syncope in patients with hypertrophic cardiomyopathy (HCM) (1, 2). Several studies have shown that a variety of ventricular and supraventricular arrhythmias (SVAs) may occur in these patients (1–10). Ventricular tachycardia (VT) is the most commonly recorded fatal arrhythmic event for sudden cardiac death (1, 2). Among all types of SVAs in HCM, atrial fibrillation (AF) has been the most extensively investigated because it affects a large proportion of these patients, involves a high risk of stroke, and is often poorly tolerated (1, 2, 11–23). For rhythm control in patients with HCM and AF, catheter ablation can be beneficial in some patients who have drug refractory symptoms or who are unable to take anti-arrhythmic drugs. However, the success rate of catheter ablation varies among different studies (1, 2).

The prevalence and characteristics of other types of SVAs have not been as well-studied as those for AF. Different types of SVAs, including atrial flutter (AFL), atrial tachycardia (AT), atrioventricular nodal reentrant tachycardia (AVNRT), and atrioventricular reentrant tachycardia (AVRT), were first identified in patients with HCM in several electrophysiological studies in the 1980s (8, 24–26). Interestingly, relatively few studies have evaluated catheter ablation of these SVAs (27–29). The current guidelines recommend that SVAs other than AF in patients with HCM should be ablated if there is an ablatable substrate (2). However, to date, the long-term ablation outcomes of non-AF SVAs have not been reported.

The understanding of HCM has improved in the modern era, but arrhythmias in patients with HCM have not been well-studied in China. Therefore, this study aimed to comprehensively investigate the characteristics and long-term ablation outcomes of AF and non-AF SVAs in a Chinese population with HCM.

## Materials and Methods

### Study Population

Between May 2010 and October 2020, 101 consecutive symptomatic patients with HCM were referred to the Arrhythmia Center, Fuwai Hospital, Beijing, for electrophysiology and arrhythmia service. The referral reasons included symptoms of palpitations, syncope or presyncope, and with or without abnormal electrocardiographic (ECG) or 24-h Holter monitoring. This study was performed in accordance with the Declaration of Helsinki and was approved by the Institutional Review Board and Ethics Committee. Informed consent was obtained from all participants.

### Diagnosis of HCM

HCM was defined by a wall thickness ≥ 15 mm in one or more left ventricular myocardial segments measured by echocardiography and/or cardiovascular magnetic resonance imaging, which was not explained solely by loading conditions (1, 2). In this study, all patients were evaluated by echocardiography and 44 (43.6%) were also evaluated by cardiovascular magnetic resonance. Greatest thickness measured at any site in the left ventricular wall was considered as the maximal thickness. In patients with intensive physical training, hypertension, valve diseases, or lesser degrees of wall thickening (13-14 mm), the diagnosis was made by at least two experts from the Cardiomyopathy Center (1, 2). In this study, seven patients had a maximal wall thickness of 13-14 mm. Left ventricular outflow tract obstruction was defined as an instantaneous peak Doppler left ventricular outflow tract pressure gradient of ≥30 mmHg (1, 2). A gradient ≥50 mmHg was considered to be hemodynamically significant (1, 2). HCM was divided into three types according to the site of the hypertrophic segment: interventricular septum only, apex only, and multi-segment (≥2 segments of the left ventricular wall).

### Diagnosis of SVAs

SVAs included in this study were AF, AFL, AT, AVRT, and AVNRT. Sinus tachycardia was not included. An initial diagnosis of arrhythmia was made by ECG and Holter monitoring in all patients. The minimum duration to establish the diagnosis of clinical and symptomatic SVA was at least 30 s, or entire 12-lead ECG. In those who underwent an electrophysiological study (EPS), a more precise diagnosis was established. The EPS-diagnosed population included 16 (32%) of the 50 AF, 7 (44%) of the 16 AFL, 3 (27%) of the 11 AT, 10 (67%) of the 15 AVRT, and 3 (100%) of the 3 AVNRT. Other patients were diagnosed on the basis of ECG and Holter after discussion between at least two electrophysiologists.

Ventricular tachyarrhythmias and bradyarrhythmias were also recorded. Ventricular tachyarrhythmias included VT and frequent premature ventricular contraction (defined as ≥ 700 per 24 h). Bradyarrhythmias included sinus node dysfunction and atrioventricular block. Atrioventricular block included first-degree, second-degree, and third-degree atrioventricular block.

### EPS and Catheter Ablation

Anti-arrhythmic drugs were discontinued five half-lives or more before EPS and catheter ablation. AF catheter ablation was performed in patients with multiple symptomatic episodes of AF in whom anti-arrhythmic drugs failed to control the frequency of AF or in whom anti-arrhythmic drugs were not tolerated. AF catheter ablation mainly consisted of antral pulmonary vein isolation by radiofrequency ablation or cryoablation. An EPS was performed in patients who were suspected of having other types of SVAs. Radiofrequency ablation was performed after initiating SVAs by routine programed stimulation.

### Follow-Up

Patients who underwent ablation were followed up by out-patient visits or telephone calls in 3 months, 6 months, 12 months, and every 1 year thereafter. All-cause death, cardiac death, stroke, and recurrence of tachycardia were recorded. The final census date for this study was January 15, 2021.

### Statistical Analysis

Continuous variables are expressed as mean ± standard deviation (SD) or median [interquartile range (IQR)] as appropriate, and categorical parameters are showed as ratio or percentage. For continuous data, either student *t*-test or Mann-Whiney *U*-test was conducted between two independent samples as appropriate. For comparisons of continuous data among multiple groups, analysis of variance or non-parametric analyses were used when the assumption of normality is in doubt. Chi-square test was used for categorical data. Receiver operative characteristic curves were used to determine the best cutoff values of predictors. Kaplan-Meier curve was carried out and log rank *p*-value was calculated in survival analysis. A *p*-value of < 0.05 was considered statistically significant. Data analyses were performed using R version 4.0.2.

## Results

### The Spectrum of Arrhythmias in the Overall Population

**Figure 1:**
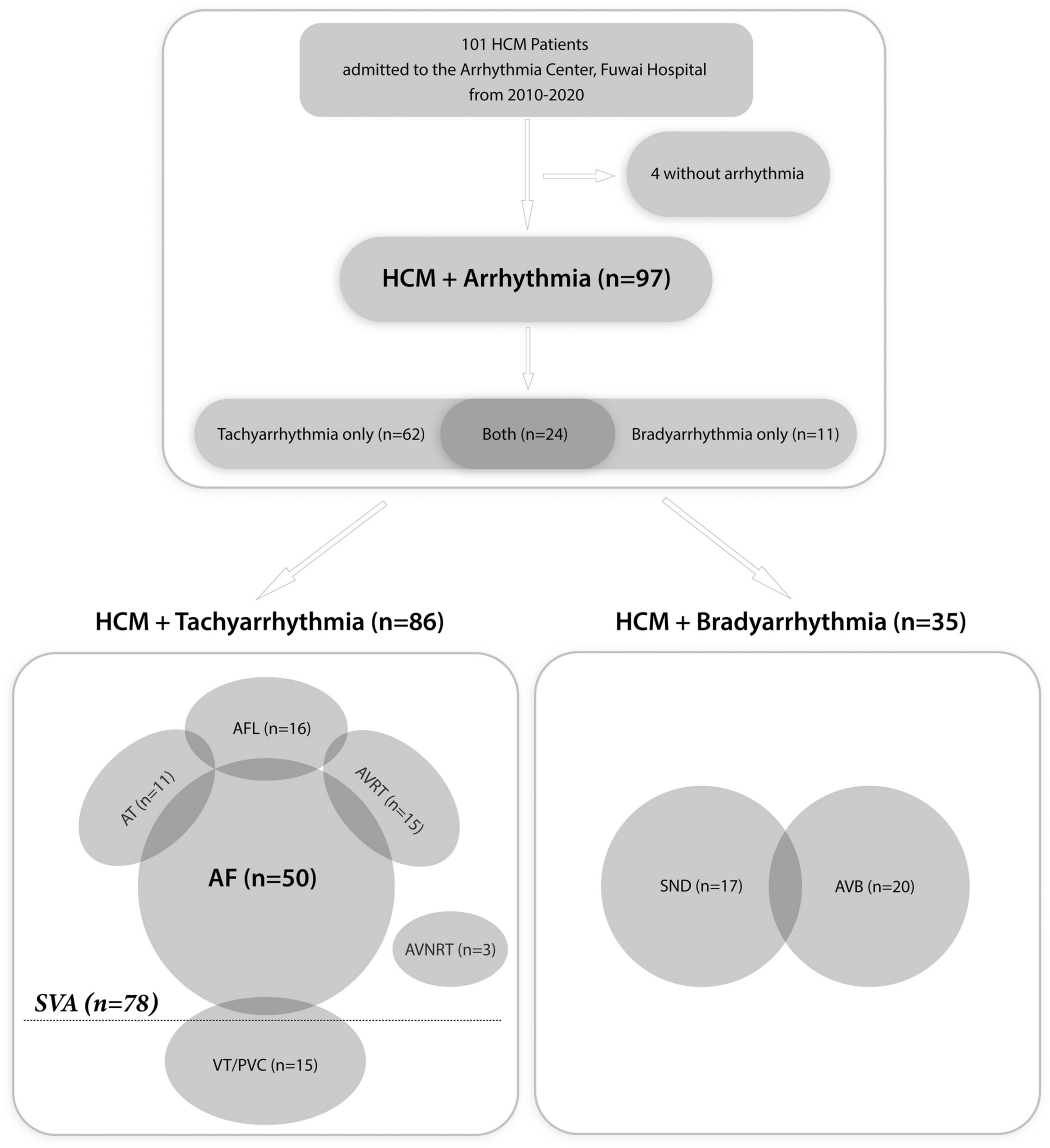
Flow chart of all patients with arrhythmias. HCM, hypertrophic cardiomyopathy; SVA, supraventricular tachyarrhythmia; AF, atrial fibrillation; AFL, atrial flutter; AT, atrial tachycardia; AVRT, atrioventricular reentrant tachycardia; AVNRT, atrioventricular nodal reentrant tachycardia; VT, ventricular tachycardia; PVC, premature ventricular contraction; SND, sinus node dysfunction; AVB, atrioventricular block.

Of all 101 patients, 4 had no evidence of arrhythmia. In the overall cohort with arrhythmia, 63 (65%) patients were male, and the median (IQR) age at admission was 58 (23.5) years old (Supplementary Table 1). Figure 1 displays the spectrum of all arrhythmias. Of the 97 patients diagnosed with arrhythmia, 86 (88.7%) had tachyarrhythmias and 35 (36.1%) had bradyarrhythmias. Specifically, 62 (63.9%) patients only had tachyarrhythmias, 11 (11.3%) only had bradyarrhythmias, and 24 (24.7%) had both. Of the 35 patients with bradyarrhythmias, 15 (42.9%) only had sinus node dysfunction, 18 (51.4%) only had atrioventricular block, and two (5.7%) had both. Of the 86 patients with tachyarrhythmias, 15 (17.4%) had VT or frequent premature ventricular contraction and 78 (90.7%) had SVAs. The clinical features of all patients with arrhythmias are shown in Supplementary Table 1.

### Baseline Characteristics of Patients With SVAs

In the SVA population (*n* = 78), 50 (64.1%) patients had AF, 16 (20.5%) had AFL, 15 (19.2%) had AVRT, 11 (14.1%) had AT, and three (3.8%) had AVNRT (Figure 1). Table 1 presents the characteristics of the SVA population. No comparisons were performed because of the relatively small size in each subgroup. There was a male preponderance in the overall SVA population and all of the subgroups, except for the AT group. The median (IQR) age at onset of arrhythmia-related symptoms and age at admission in the overall SVA population were 55 (27) and 59 (23) years, respectively. Patients with AVRT, AVNRT, or AFL appeared to be younger, and patients with AF or AT were older at the onset of arrhythmia-related symptoms and at admission. A history of syncope was reported in 15% (19.2%) of all patients. More than half of the patients with AT had syncope. A positive family history of HCM was recorded in eight (6.4%) patients.

**Table 1:** Demographic and clinical characteristics.

**Parameters**	**All SVA** **(*n* = 78)**	**AF** **(*n* = 50)**	**AFL** **(*n* = 16)**	**AT** **(*n* = 11)**	**AVRT** **(*n* = 15)**	**AVNRT** **(*n* = 3)**
Male gender, *n* (%)	50 (64.1)	26 (52)	12 (75)	4 (36.4)	11 (73.3)	3 (100)
Weight, kg	70 ± 14.9	71 ± 14.3	68 ± 15.7	67 ± 18.6	64 ± 14.5	82 ± 7.9
Height, cm	169 ± 8.3	168 ± 9.2	169 ± 7.1	164 ± 9.5	169 ± 6.5	174 ± 6.0
BSA, m^2^	1.79 ± 0.21	1.79 ± 0.22	1.76 ± 0.21	1.71 ± 0.26	1.73 ± 0.19	1.96 ± 0.10
BMI, kg/m^2^	24.6 ± 4.19	25.1 ± 3.86	23.5 ± 4.53	24.7 ± 4.72	22.5 ± 4.31	27.3 ± 3.51
Age at admission, y	59 (23)	63 (21)	43 (25)	66 (14)	48 (30)	43 (NA)
Age at arrhythmia symptom onset, y	55 (27)	59 (23)	38 (29)	63 (23)	36 (38)	43 (NA)
Arrhythmia symptoms coincided with SVA events on Holter, *n* (%)	69 (88)	43 (86)	16 (100)	9 (82)	15 (100)	3 (100)
Age at HCM symptom onset, y	51 (29)	58 (23)	35 (31)	62 (25)	32 (28)	43 (NA)
History of syncope, *n* (%)	15 (19.2)	7 (14)	4 (25)	6 (54.5)	2 (13.3)	0 (0)
Family history of HCM, *n* (%)	5 (6.4)	4 (8)	1 (6.3)	0 (0)	0 (0)	0 (0)
SCD risk score, %	2.24 ± 1.54	2.13 ± 1.63	3.09 ± 1.49	2.39 ± 1.74	1.85 ± 0.96	1.56 ± 0.19
ICD implantation, *n* (%)	2 (2.6)	2 (4.0)	0 (0)	1 (9.1)	0 (0)	0 (0)
Comorbidities, *n* (%)	36 (46.2)	24 (48)	8 (50)	5 (45.5)	3 (20)	2 (66.7)
HTN, *n* (%)	31 (39.7)	20 (40)	4 (25)	7 (63.6)	7 (46.7)	1 (33.3)
CAD, *n* (%)	14 (17.9)	9 (18)	2 (12.5)	3 (27.3)	1 (6.7)	1 (33.3)
DM, *n* (%)	13 (16.7)	10 (20)	1 (6.3)	2 (18.2)	2 (13.3)	1 (33.3)
CHD, *n* (%)	3 (3.8)	3 (6)	1 (6.3)	0 (0)	0 (0)	0 (0)
VHD, *n* (%)	2 (2.6)	2 (4)	1 (6.3)	0 (0)	0 (0)	0 (0)
PH, *n* (%)	8 (10.3)	7 (14)	2 (12.5)	0 (0)	0 (0)	0 (0)
History of stroke, *n* (%)	8 (10.3)	4 (8)	4 (25)	2 (18.2)	1 (6.7)	0 (0)
NYHA-FC, *n* (%)						
I/II	71 (91.0)	46 (92)	15 (92.7)	11 (63.6)	15 (100)	15 (100)
III/IV	7 (9.0)	4 (8)	1 (6.3)	4 (36.4)	0 (0)	0 (0)

*SVA, supraventricular tachyarrhythmia; AF, atrial fibrillation; AFL, atrial flutter; AT, atrial tachycardia; AVRT, atrioventricular reentrant tachycardia; AVNRT, atrioventricular nodal reentrant tachycardia; BSA, body surface area; BMI, body mass index; HCM, hypertrophic cardiomyopathy; HTN, hypertension; CAD, coronary artery disease; DM, diabetes mellitus; CHD, congenital heart disease; VHD, valvular heart disease; PH, pulmonary hypertension; NYHA-FC, New York Heart Association functional class*.

Comorbid disorders were recorded in 36 (46.2%) patients. Hypertension was the most frequent (39.7%) comorbid disease, followed by coronary artery disease (17.9%) and diabetes mellitus (16.7%). Pulmonary hypertension was observed by echocardiography in eight (10.3%) patients. The rate of hypertension was more frequent in patients with AT than those in other patients. The New York Heart Association functional class was evaluated in all of the patients at admission. Most patients were in functional class I/II (91%). The AT group appeared to have more patients in functional class III/IV than other subgroups.

Table 2 shows the baseline echocardiography parameters. The multi-segment (50%) and interventricular septum (42.3%) comprised the majority of hypertrophic types, and only six (7.7%) involved the apex. The median (IQR) maximum left ventricular wall thickness was 19 (6) mm in all patients and tended to be smaller in the AVRT group than in the other subgroups. More patients in the AT subgroup had left ventricular outflow tract obstruction than those in the other subgroups. Most patients appeared to have a normal systolic function with a median (IQR) left ventricular ejection fraction of 63% (10.3), a normal left ventricular end-diastolic dimension (LVEDD) of 45 (8.3) mm, and a greater left atrial dimension of 41 (11) mm. The left ventricular ejection fraction and LVEDD appeared to be similar among the subgroups. Patients with AFL had the greatest left atrial dimension, while the AVNRT group had the smallest.

**Table 2:** Echocardiography parameters.

**Parameters**	**All SVA** **(*n* = 78)**	**AF** **(*n* = 50)**	**AFL** **(*n* = 16)**	**AT** **(*n* = 11)**	**AVRT** **(*n* = 15)**	**AVNRT** **(*n* = 3)**
HCM types						
IVS hypertrophy	33 (42.3)	21 (42)	6 (37.5)	4 (36.4)	6 (40)	1 (33.3)
Apex hypertrophy	6 (7.7)	5 (10)	0 (0)	0 (0)	1 (6.7)	0 (0)
Multi-segment hypertrophy	39 (50)	24 (48)	10 (62.5)	7 (63.6)	8 (53.3)	2 (66.7)
LVOT obstruction, *n* (%)	19 (24.4)	13 (26)	2 (12.5)	5 (45.5)	3 (20)	0 (0)
Ejection fraction, %	63 (10.3)	63 (11.5)	61 (6.5)	65 (10.0)	65 (11.0)	60 (NA)
LA dimension (AP), mm	41 (11.0)	43 (8.5)	45 (13.8)	39 (15.0)	38 (10.0)	25 (NA)
LVEDD, mm	45 (8.3)	45 (8.0)	44 (13.8)	45 (9.0)	43 (9.0)	46 (NA)
Max LV thickness, mm	19 (6)	19 (5.3)	23 (6.50)	19 (4.0)	16 (4.0)	25 (NA)
Mitral regurgitation ≥ moderate, *n* (%)	9 (11.6)	6 (12)	2 (12.5)	1 (9.1)	1 (6.7)	0 (0)

*SVA, supraventricular tachyarrhythmia; AF, atrial fibrillation; AFL, atrial flutter; AT, atrial tachycardia; AVRT, atrioventricular reentrant tachycardia; AVNRT, atrioventricular nodal reentrant tachycardia; HCM, hypertrophic cardiomyopathy; IVS, interventricular septum; LVOT, left ventricular outflow tract; ECHO, echocardiography; LA, left atrial; AP, anteroposterior; LVEDD, left ventricular end-diastolic dimension; LV, left ventricle*.

Medical treatment information was obtained in all patients (Supplementary Table 2). Beta-blockers and non-dihydropyridine-calcium channel blockers were used in 63 (81%) patients to improve symptoms of HCM. Other patients were not treated with these drugs because of the presence of bradyarrhythmia. The most frequently used anti-arrhythmic drugs were amiodarone (24.4%) and sotalol (10.3%).

Comparisons between persistent AF and paroxysmal AF are shown in Supplementary Table 3. Patients with persistent AF were heavier (*p* = 0.017) and had a greater body surface area (*p* = 0.022) than patients with PAF. The echocardiographic parameters and functional class were similar in the two groups. Significantly more patients with persistent AF used warfarin (*p* = 0.036) for anticoagulation than those with paroxysmal AF. More patients with paroxysmal AF used amiodarone for rhythm control than those with persistent AF (*p* = 0.043).

### Catheter Ablation for SVAs

Among the patients with SVAs, 43 were scheduled for catheter ablation. Eventually, ablation was not performed in eight patients and failed in one patient. Two patients scheduled for AF ablation were not ablated. One of these patients was not ablated because of acute heart failure pre-procedure and one because of failure of placing a coronary sinus electrode and atrial transseptal puncture. Tachycardia was not initiated in three patients who were suspected of having AVNRT or AVRT, and in three patients who were suspected of having AT. Ablation was terminated in one patient with AF because of acute injury to the phrenic nerve.

**Figure 2:**
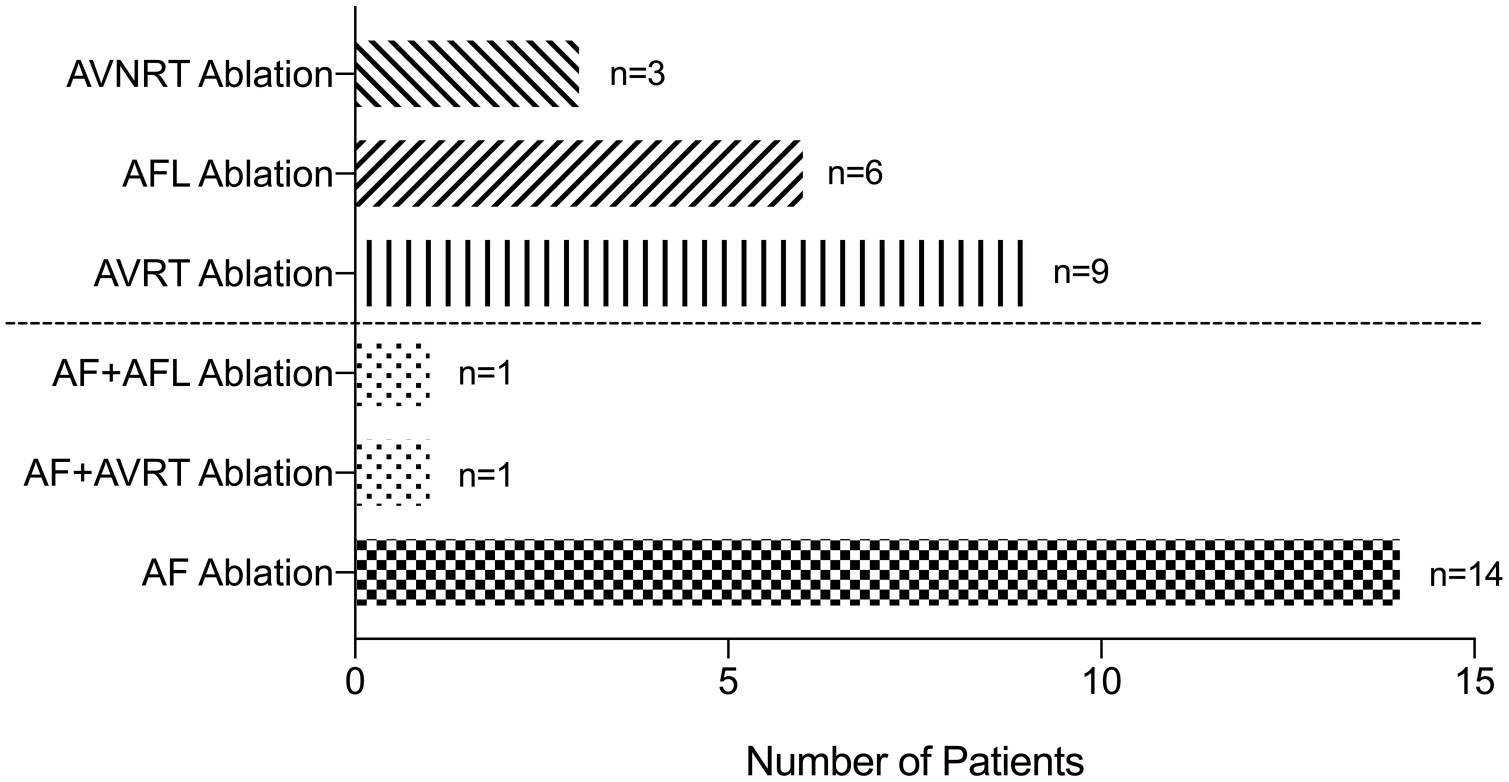
Patients underwent successful ablation. AF, atrial fibrillation; AFL, atrial flutter; AT, atrial tachycardia; AVRT, atrioventricular reentrant tachycardia; AVNRT, atrioventricular nodal reentrant tachycardia.

Finally, 34 patients underwent catheter ablation successfully, and 16 of them had AF (Figure 2). Of the remaining 18 patients, 9 had AVRT, 6 had AFL, and 3 had AVNRT. One patient was ablated for both AF and AVRT, and one was ablated for both AF and AFL. Of the 16 patients who had AF ablation, 4 had persistent AF and 12 had paroxysmal AF. Three of these patients were ablated with the cryoablation method and 13 with the radiofrequency ablation method. The other 18 patients with non-AF SVAs were ablated with the radiofrequency ablation method. The characteristics of patients who underwent catheter ablation are shown in Supplementary Table 4.

### Outcomes and Survival Analysis

**Figure 3:**
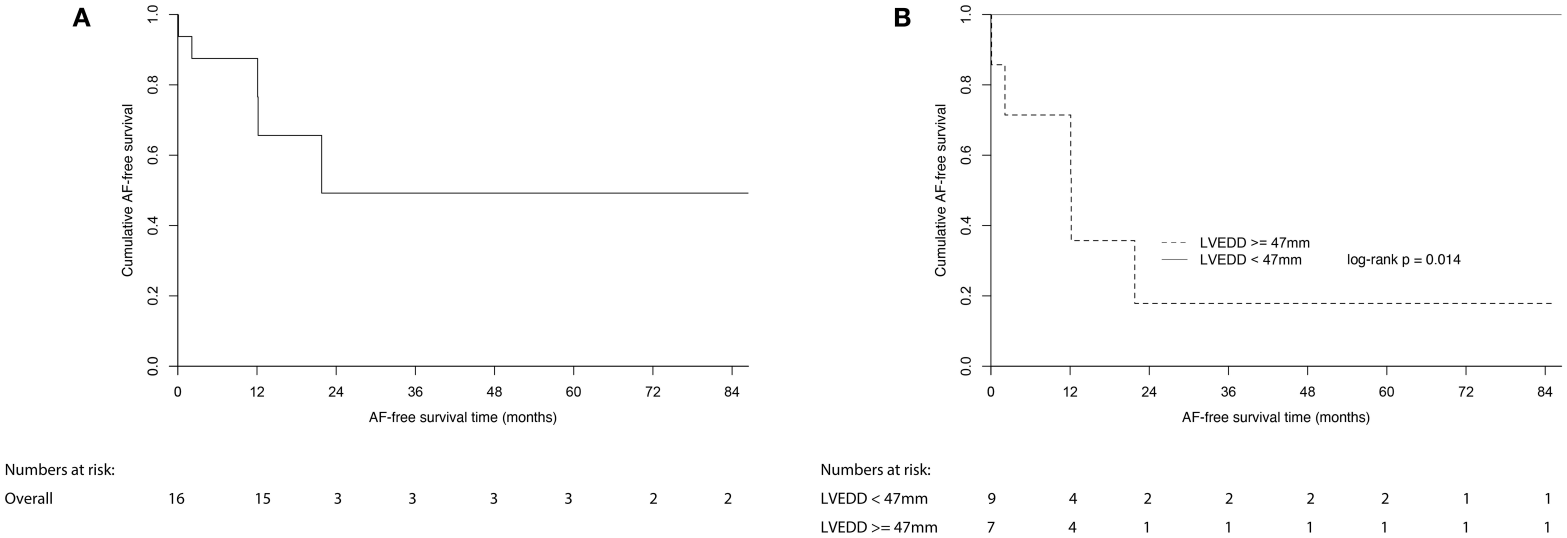
Estimated AF-free survival of **(A)** all patients with AF underwent ablation; **(B)** patients with AF underwent ablation divided by LVEDD of 47 mm. AF, atrial fibrillation; LVEDD, left ventricular end-diastolic dimension.

Patients who underwent ablation were followed up for a median (IQR) of 58.5 (82.9) months, and no death or stroke event occurred. There was no recurrence in the 18 patients who were ablated for other types of SVAs. Five patients who were ablated for AF experienced recurrence. A survival analysis using the Kaplan–Meier curve method in patients who were ablated for AF is shown in Figure 3. The 1- and 7-year AF-free survival rates were 87.5 and 49.2%, respectively (Figure 3A). The AF-free survival was similar in patients with paroxysmal and persistent AF (data not shown).

**Figure 4:**
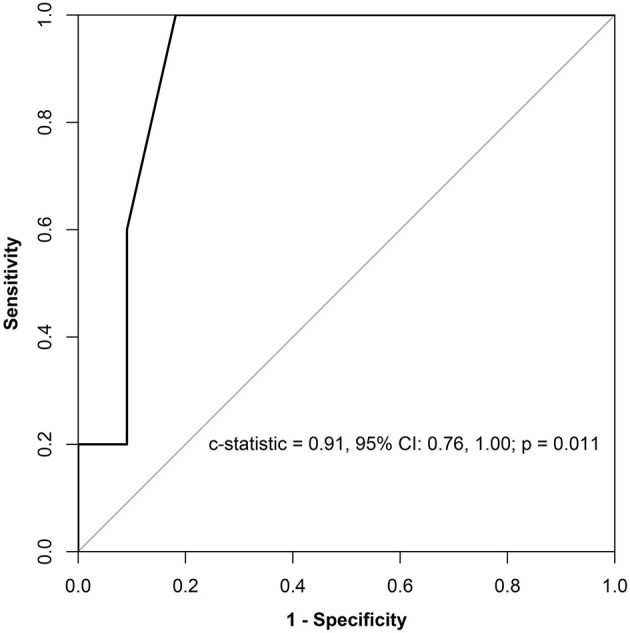
Prognostic sensitivity and specificity of LVEDD for AF recurrence. LVEDD, left ventricular end-diastolic dimension; CI, confidential interval; AF, atrial fibrillation.

All of the echocardiographic and clinical factors were compared between patients who experienced recurrences and patients who did not (Supplementary Table 5). Among all of the parameters, LVEDD was the only parameter that was significantly different between the two groups (*p* = 0.009, Supplementary Table 5). A receiver operator characteristic analysis showed that a greater LVEDD was associated with a higher recurrence of AF, with an optimum cutoff value of 47 mm (c-statistic = 0.91, *p* = 0.011, sensitivity = 1.00, specificity = 0.82) (Figure 4). In Kaplan–Meier analysis, patients with a LVEDD ≥ 47 mm had a worse AF-free survival than those with a LVEDD <47 mm (log-rank *p* = 0.014) (Figure 3B).

## Discussion

HCM has a prevalence of 1 case per 200-500 persons in the general population and affects ~20 million people globally (30). HCM has been underrecognized in third-world countries including China for many years, but an awareness of this disease is now penetrating the health care system in China (30). This study aimed to provide a comprehensive clinical profile of different types of SVAs and report the long-term outcomes of catheter ablation in a tertiary-based HCM population in China. Unlike other studies of arrhythmia in a general HCM population, the particular population in the present study were patients with HCM who were referred to the Arrhythmia Center who already had arrhythmia-related symptoms with or without an abnormal ECG indicating arrhythmia at enrollment.

In this study, among the 86 patients with tachyarrhythmia, 78 (90%) had SVAs, in whom AF was the most common, followed by AFL, AVTR, focal AT, and AVNRT. AF was the most common arrhythmia in the present population, and it accounted for 64% of SVAs, 58% of tachyarrhythmias, and 52% of arrhythmias. This prevalence is consistent with previous studies (2, 15). Of the 50 patients with AF, 7 (14%) also had AFL. This percentage is similar to that reported in a study conducted by Rowin and colleagues (15). Surprisingly, 15 (15.5%) patients had AVRT, including 5 with Wolff-Parkinson-White syndrome. Fananapazir et al. showed that accessory atrioventricular pathways were only present in seven (5%) patients and only one had a record of AVRT (8). In their study, dual atrioventricular nodal pathways were present in three patients and neither of them had records of spontaneous AVNRT. However, in our study, three (3.1%) patients had AVNRT. Additionally, 11 (11.3%) patients were diagnosed with focal AT by ECG and Holter monitoring. Except for AFL, no reentrant AT was diagnosed in this study.

The first choice of rhythm control strategy in HCM patients with AF is antiarrhythmic drugs (1, 2). Catheter ablation is also an important option, while it is less effective than in the general population (1, 2). In this study, successful catheter ablation was performed in 16 patients in whom 3 with cryoablation and the other 13 with radiofrequency ablation. The ablation strategy was pulmonary vein isolation in 13 patients with persistent AF and in two patients with persistent AF. In the other two patients with persistent AF, ablation for adjunctive lines was performed. A meta-analysis of 15 studies conducted by Zhao et al. reported a pooled single-procedure success rate of 45.5% at 18 months (14). Another meta-analysis by Providencia et al. included five studies and showed that the single-procedure success rate was 38.7% in patients with HCM (31). The success rate of AF ablation in our study was relatively high, and this finding could have occurred for the following reasons: (1) the sample size was relatively small; (2) patients who underwent catheter ablation were carefully evaluated and selected; (3) most (75%) of the patients had paroxysmal AF; and (4) some patients might have had recurrence without symptoms. Further randomized studies on AF ablation in patients with HCM are required.

There have only been a few studies on ablation in patients with HCM in other types of SVAs, including AFL, AVRT, and AVNRT. On the basis of the European Society of Cardiology guideline recommendations that EPS should be performed in patients with palpitations and the ablatable substrate for SVAs should be treated, we performed catheter ablation in 20 patients with other types of SVAs. Of the 20 patients, 1 had AVRT ablated simultaneously with AF and 1 had AFL ablated simultaneously with AF. Of the 10 patients with AVRT, 6 were left-sided and 4 were right-sided accessory pathways. All of the three AVNRTs were the slow-fast type. Of the seven AFLs ablated, five were typical cavotricuspid isthmus-dependent and two were non-cavotricuspid isthmus-dependent from the left atria. During follow-up, there was no recurrence of AFL, AVRT, or AVNRT. The success rate of catheter ablation was similar to that in the general population. The present study provides further evidence for EPS and ablation of non-AF SVAs in HCM.

In this study, we could not obtain genotype information which is important for classifying the etiology of HCM. Some etiologies might be associated with arrhythmia. Monda et al. and Limongelli et al. concluded that pre-excitation, atrioventricular block, and concentric left ventricular hypertrophy might be diagnostic clues for non-sarcomeric etiologies (32, 33).

## Limitations

Firstly, this was a retrospective analysis from a single tertiary arrhythmia center. Therefore, there was likely to have been patient selection bias. Second, genotype information was not available in this study. Future studies on the relationship between genotype and SVAs in HCM are required. Third, some patients in this study were diagnosed by ECG and Holter monitoring rather than EPS, which might not have been precise, especially in patients with AT and AVRT. However, these cases were discussed thoroughly between at least two electrophysiologists in our center. Lastly, although this was a 10-year analysis, the sample size was small. One reason for this small sample size is that HCM is not a common disease and it is underrecognized in many places in China. Another reason for this small sample size is that the general population of HCM is referred to the Cardiomyopathy Center in our hospital while the population in this study only included patients with HCM who were referred to the electrophysiology and arrhythmia service. Surprisingly, this is a unique population that has not been previously reported. Further multicenter studies of SVAs in the general HCM population are required.

## Conclusions

In this unique population of HCM, AF was the most common SVA, followed in order by AFL, AVRT, AT, and AVNRT. The long-term catheter ablation outcome for non-AF SVAs in HCM is satisfactory. A greater LVEDD predicts AF recurrence after catheter ablation in patients with HCM.

## Data Availability Statement

The datasets presented in this article are not readily available because research data is confidential. Data sharing requests are required to meet the policies of the hospital and the funder. Requests to access the datasets should be directed to doctortangmin@yeah.net.

## Ethics Statement

The studies involving human participants were reviewed and approved by Fuwai Hospital. The patients/participants provided their written informed consent to participate in this study.

## Author Contributions

H-DZ, J-TZ, P-HF, SZ, and MT: study conception and design. H-DZ and MT: analysis and interpretation of data, drafting of the article, and obtaining of funding. H-DZ, LD, S-XW, BZ, X-TD, L-XH, Y-JQ, F-YY, T-JF, J-TZ, WH, P-HF, SZ, and MT: critical revision of the article for intellectual content and final approval of the article. J-TZ, P-HF, WH, SZ, and MT: provision of study materials or patients. H-DZ, P-HF, SZ, and MT: statistical expertise. H-DZ, J-TZ, P-HF, WH, SZ, and MT: administrative, technical, or logistic support. H-DZ, LD, and S-XW: collection of data. All authors contributed to the article and approved the submitted version.

## Funding

The study was supported by the National Natural Science Foundation of China (82000064). The funding source had no role in study design; in the collection, analysis, and interpretation of data; in writing the report; and in the decision to submit the article for publication.

## Conflict of Interest

The authors declare that the research was conducted in the absence of any commercial or financial relationships that could be construed as a potential conflict of interest.

## Publisher's Note

All claims expressed in this article are solely those of the authors and do not necessarily represent those of their affiliated organizations, or those of the publisher, the editors and the reviewers. Any product that may be evaluated in this article, or claim that may be made by its manufacturer, is not guaranteed or endorsed by the publisher.
