# Supplementary material for: Characteristics and Long-Term Ablation Outcomes of Supraventricular Arrhythmias in Hypertrophic Cardiomyopathy: A 10-Year, Single-Center Experience
Source: Front Cardiovasc Med. 2021 Nov 12;8:766571. doi: 10.3389/fcvm.2021.766571 (PMC8632865; doi:10.3389/fcvm.2021.766571)
Supplement: Supplementary file 1 [file Data_Sheet_1.docx]

**SUPPLEMENTAL MATERIAL**

**Supplemental Table 1. Demographic and clinical characteristics in all patients with arrhythmia**

| **Parameters** | **Overall**  **(n = 97)** | **TACHY-only**  **(n = 62)** | **BRADY-only**  **(n = 11)** | **TACHY+BRADY**  **(n = 24)** | **P value** |
| --- | --- | --- | --- | --- | --- |
| Male gender, n(%) | 63(65) | 38(61) | 5(45) | 18(75) | 0.222 |
| Weight, Kg | 70 ± 14.4 | 72 ± 15.4 | 62 ± 10.4 | 67 ± 11.9 | 0.086 |
| Height, cm | 168 ± 8.2 | 169 ± 8.5 | 165 ± 8.7 | 169 ± 6.9 | 0.319 |
| BSA, m^2^ | 1.78 ± 0.20 | 1.81 ± 0.22 | 1.67 ± 0.15 | 1.76 ± 0.17 | 0.115 |
| BMI, kg/m^2^ | 24.4 ± 4.16 | 25.0 ± 4.31 | 23.1 ± 4.25 | 23.5 ± 3.44 | 0.144 |
| Age at admission, y | 58(23.5) | 56(22.3) | 45(21.0) | 64(26.8) | 0.012 |
| Age at arrhythmia symptom onset, y | 51(26.0) | 52(26.0) | 45(19.0) | 58(25.0) | 0.231 |
| Age at HCM symptom onset, y | 47(28.7) | 47(28.9) | 38 (10.9) | 57(30.2) | 0.104 |
| SCD Risk Score, % | 2.52 ± 1.88 | 2.44 ± 2.06 | 3.11 ± 1.53 | 2.45 ± 1.51 | 0.542 |
| ICD Implantation, n(%) | 5(5.2) | 5(8.1) | 0(0) | 0(0) | NA |
| HCM types |  |  |  |  | 0.024 |
| IVS hypertrophy | 44(45.4) | 29(46.8) | 9(81.8) | 6(25) |  |
| Apex hypertrophy | 8(8.2) | 4(6.5) | 0(0) | 4(16.7) |  |
| Multi-segment hypertrophy | 45(46.4) | 29(46.8) | 2(18.2) | 14(58.3) |  |
| LVOT Obstruction, n(%) | 25(26) | 13(21) | 6(55) | 6(25) | 0.063 |
| History of syncope, n(%) | 23(23) | 8(13) | 3(27) | 12(50) | 0.001 |
| Family history of HCM, n(%) | 8(8) | 7(11) | 1(9) | 0(0) | 0.232 |
| ECHO parameters |  |  |  |  |  |
| Ejection fraction, % | 63(10.0) | 63(12.1) | 65(14.0) | 64(7.5) | 0.733 |
| LA dimension (AP), mm | 41(10) | 42(11.5) | 42(4.0) | 41(9.5) | 0.717 |
| LVEDD, mm | 45 ± 6.7 | 44 ± 6.5 | 46 ± 7.6 | 46 ± 7.0 | 0.409 |
| Max LV thickness, mm | 19(7.0) | 19(5.5) | 22(9.0) | 19(6.8) | 0.261 |
| Mitral regurgitation ≥moderate, n(%) | 13(13) | 7(11) | 3(27) | 3(13) | 0.305 |
| Comorbidities | 44(45.4) | 29(46.8) | 3(27.3) | 12(50) | 0.425 |
| HTN, n(%) | 37(38) | 23(37) | 1(9) | 13(54) | 0.037 |
| CAD, n(%) | 18(19) | 10(16) | 1(9) | 7(29) | 0.262 |
| DM, n(%) | 13(13) | 10(16) | 0(0) | 3(13) | 0.347 |
| CHD, n(%) | 3(3) | 3(5) | 0(0) | 0(0) | 0.417 |
| VHD, n(%) | 2(2) | 2(3) | 0(0) | 0(0) | 0.562 |
| PH, n(%) | 11(11) | 7(11) | 2(18) | 2(8) | 0.695 |
| History of Stroke, n(%) | 9(9) | 8(13) | 0(0) | 1(4) | 0.242 |
| NYHA-FC, n(%) |  |  |  |  | 0.493 |
| I/II | 86(89) | 57(92) | 8(73) | 21(87) |  |
| III/IV | 11(11) | 5(8) | 3(27) | 3(13) |  |
| Medications |  |  |  |  |  |
| HCM symptom improving therapy, n(%) |  |  |  |  |  |
| Beta-blockers | 62(64) | 39(63) | 7(64) | 16(67) | 0.948 |
| Non-DHP-CCB | 15(15) | 11(18) | 1(9) | 3(13) | 0.688 |
| Antiarrhythmia drugs, n(%) |  |  |  |  |  |
| Amiodarone | 23(24) | 18(29) | 0(0) | 5(21) | 0.105 |
| Dronedarone | 1(1) | 1(2) | 0(0) | 0(0) | 0.752 |
| Propafenone | 3(3) | 2(3) | 0(0) | 1(4) | 0.800 |
| Sotalol | 8(8) | 7(11) | 0(0) | 1(4) | 0.321 |
| Anticoagulants, n(%) |  |  |  |  |  |
| Warfarin | 24(25) | 18(29) | 1(9) | 5(21) | 0.324 |
| Rivaroxaban | 6(6) | 6(10) | 0(0) | 0(0) | 0.164 |
| Dabigatran | 8(8) | 8(13) | 0(0) | 0(0) | 0.085 |
| Other Drug therapy, n(%) |  |  |  |  |  |
| Asprin | 30(30) | 20(32) | 1(9) | 9(38) | 0.224 |
| Clopidogrel | 7(7) | 4(6) | 0(0) | 3(13) | 0.385 |
| ACEI/ARB | 31(32) | 19(31) | 2(18) | 10(42) | 0.359 |
| Alpha-blockers | 6(6) | 5(8) | 1(9) | 0(0) | 0.347 |
| CCB | 16(16) | 10(16) | 0(0) | 6(25) | 0.179 |
| Diuretics | 25(26) | 14(23) | 4(36) | 7(29) | 0.571 |
| MRA | 14(14) | 8(13) | 2(18) | 4(17) | 0.844 |
| Statins | 33(34) | 20(32) | 3(27) | 10(42) | 0.627 |
| Antidiabetics | 12(12) | 9(15) | 0(0) | 3(13) | 0.403 |

Definitions of abbreviations: TACHY, tachyarrhythmia; BRADY, bradyarrhythmia; BSA, body surface area; BMI, body mass index; HCM, hypertrophic cardiomyopathy; IVS, interventricular septum; LVOT, left ventricular outflow tract; ECHO, echocardiography; LA, left atrial; AP, anteroposterior ; LVEDD, left ventricular end-diastolic dimension; LV, left ventricle; HTN, hypertension; CAD, coronary artery disease; DM, diabetes mellitus; CHD, congenital heart disease; VHD, valvular heart disease; PH, pulmonary hypertension; NYHA-FC, New York Heart Association functional class; Non-DHP, Non-dihydropyridine; CCB, calcium channel blocker; ACEI, angiotensin converting enzyme inhibitor; ARB, angiotensin receptor blocker; MRA, mineralocorticoid receptor antagonist.

Values are expressed as n (%), ratio, mean ± SD, or median (interquartile range).

* Comparison among patients in the three groups of tachyarrhythmia only, bradyarrhythmia only, and tachyarrhythmia + bradyarrhythmia, analysis of variance (ANOVA) or non-parametric analyses for continuous data, and chi-square test for categorical data.

**Supplemental Table 2. Medications at discharge for patients with SVA**

| **Parameters** | **All SVA**  **(n = 78)** | **AF**  **(n = 50)** | **AFL**  **(n = 16)** | **AT**  **(n = 11)** | **AVRT**  **(n = 15)** | **AVNRT**  **(n = 3)** |
| --- | --- | --- | --- | --- | --- | --- |
| HCM symptom improving therapy, n(%) |  |  |  |  |  |  |
| Beta-blockers | 49(62.8) | 33(66) | 8(50) | 9(81.8) | 7(46.7) | 0(0) |
| Non-DHP-CCB | 14(17.9) | 12(24) | 3(18.8) | 2(18.2) | 0(0) | 0(0) |
| Antiarrhythmia drugs, n(%) |  |  |  |  |  |  |
| Amiodarone | 19(24.4) | 17(34) | 4(25) | 3(27.3) | 1(6.7) | 0(0) |
| Dronedarone | 1(1.3) | 1(2) | 0(0) | 0(0) | 00(0) | 0(0) |
| Propafenone | 3(3.8) | 3(6) | 0(0) | 2(18.2) | 00(0) | 0(0) |
| Sotalol | 8(10.3) | 6(12) | 2(12.5) | 2(18.2) | 1(13.3) | 0(0) |
| Anticoagulants, n(%) |  |  |  |  |  |  |
| Warfarin | 22(28.2) | 19(38) | 3(18.8) | 4(36.4) | 1(6.7) | 0(0) |
| Rivaroxaban | 6(7.7) | 6(12) | 0(0) | 1(9.1) | 1(6.7) | 0(0) |
| Dabigatran | 8(10.3) | 7(14) | 2(12.5) | 0(0) | 0(0) | 0(0) |
| Other Drug therapy, n(%) |  |  |  |  |  |  |
| Asprin | 26(33.3) | 9(18) | 5(31.3) | 5(45.5) | 10(66.7) | 0(0) |
| Clopidogrel | 6(7.7) | 3(6) | 1(6.3) | 1(9.1) | 2(6.7) | 1(33.3) |
| ACEI/ARB | 23(29.5) | 17(34) | 3(18.8) | 4(36.4) | 4(26.7) | 1(33.3) |
| Alpha-blockers | 4(5.1) | 3(6) | 0(0) | 0(0) | 1(6.7) | 0(0) |
| CCB | 14(17.9) | 9(18) | 2(12.5) | 4(36.4) | 4(26.7) | 0(0) |
| Diuretics | 19(24.4) | 14(28) | 4(25) | 3(27.3) | 1(6.7) | 0(0) |
| MRA | 11(14.1) | 10(20) | 1(6.3) | 2(18.2) | 0(0) | 0(0) |
| Statins | 24(30.8) | 15(3) | 4(25) | 6(54.5) | 4(26.7) | 1(33.3) |
| Antidiabetics | 12(15.4) | 9(18) | 0(0) | 2(18.2) | 2(13.3) | 1(33.3) |

Definitions of abbreviations: SVA, supraventricular tachyarrhythmia; AF, atrial fibrillation; AFL, atrial flutter; AT, atrial tachycardia; AVRT, atrioventricular reentrant tachycardia; AVNRT, atrioventricular nodal reentrant tachycardia; Non-DHP, Non-dihydropyridine; CCB, calcium channel blocker; ACEI, angiotensin converting enzyme inhibitor; ARB, angiotensin receptor blocker; MRA, mineralocorticoid receptor antagonist.

Values are expressed as n (%), ratio, mean ± SD, or median (interquartile range).

**Supplemental Table 3. Demographic and clinical characteristics in all patients with AF**

| **Parameters** | **All AF patients**  **(n = 50)** | **Paroxysmal**  **(n = 35)** | **Persistent**  **(n = 15)** | **P value*** |
| --- | --- | --- | --- | --- |
| Male gender, n(%) | 26(52) | 16(45.7) | 10(66.7) | 0.174 |
| Weight, kg | 71 ± 14.3 | 68 ± 14.1 | 78 ± 12.5 | 0.017 |
| Height, cm | 168 ± 9.2 | 166 ± 8.9 | 171 ± 9.5 | 0.099 |
| BSA, m^2^ | 1.79 ± 0.22 | 1.74 ± 0.21 | 1.90 ± 0.21 | 0.022 |
| BMI, kg/m^2^ | 25.1 ± 3.86 | 24.5 ± 4.08 | 26.7 ± 2.84 | 0.059 |
| Age at admission, y | 59 ± 13.7 | 59 ± 14.0 | 60 ± 13.5 | 0.831 |
| Age at arrhythmia symptom onset, y | 59(23.0) | 57(24.0) | 59(14.0) | 0.853 |
| Age at arrhythmia diagnosis, y | 60(18.0) | 61(28.0) | 59(12.0) | 0.665 |
| HCM types |  |  |  | 0.728 |
| IVS | 21(42) | 14(40) | 7(46.7) |  |
| Apex | 5(10) | 3(8.6) | 2(13.3) |  |
| Multi-segment | 24(48) | 18(51.4) | 6(40) |  |
| LVOT Obstruction, n(%) | 13(26) | 10(28.6) | 3(20) | 0.527 |
| History of syncope, n(%) | 7(14) | 5(14.3) | 2(13.3) | 0.929 |
| Family history of HCM, n(%) | 4(8) | 2(5.7) | 2(13.3) | 0.363 |
| ECHO parameters |  |  |  |  |
| Ejection fraction, % | 62 ± 8.4 | 62 ± 6.9 | 63 ± 11.4 | 0.721 |
| LA dimension (AP), mm | 43(8.5) | 43(9.0) | 45(9.0) | 0.422 |
| LVEDD, mm | 44 ± 5.4 | 44 ± 5.5 | 45 ± 5.2 | 0.295 |
| Max LV thickness, mm | 19 ± 4.0 | 19 ± 3.6 | 20 ± 4.8 | 0.221 |
| Mitral regurgitation ≥moderate, n(%) | 6(12) | 5(14.3) | 1(6.7) | 0.291 |
| Comorbidities | 24(48) | 8(53.3) | 16(45.7) | 0.621 |
| HTN, n(%) | 20(40) | 15(42.9) | 5(33.3) | 0.529 |
| CAD, n(%) | 9(18) | 8(22.9) | 1(6.7) | 0.172 |
| DM, n(%) | 10(20) | 6(17.1) | 4(26.7) | 0.440 |
| CHD, n(%) | 3(6) | 3(8.6) | 0(0) | 0.242 |
| VHD, n(%) | 2(4) | 1(2.9) | 1(6.7) | 0.529 |
| PH, n(%) | 7(14) | 4(11.4) | 3(20) | 0.423 |
| History of Stroke, n(%) | 4(8) | 2(5.7) | 2(13.3) | 0.363 |
| NYHA-FC, n(%) |  |  |  | 0.331 |
| I/II | 46(92) | 32(91.4) | 14(93.3) |  |
| III/IV | 4(8) | 3(8.6) | 1(6.7) |  |
| Medications |  |  |  |  |
| HCM symptom improving therapy |  |  |  |  |
| Beta-blockers | 33(66) | 24(68.6) | 9(60.0) | 0.558 |
| Non-DHP-CCB | 12(24) | 8(22.9) | 4(26.7) | 0.773 |
| Antiarrhythmia drugs, n(%) |  |  |  |  |
| Amiodarone | 17(34) | 15(42.9) | 2(13.3) | 0.043 |
| Dronedarone | 1(2) | 0(0) | 1(6.7) | 0.123 |
| Propafenone | 3(6) | 3(8.6) | 0(0) | 0.242 |
| Sotalol | 6(12) | 5(14.3) | 1(6.7) | 0.447 |
| Anticoagulants, n(%) |  |  |  |  |
| Warfarin | 19(38) | 10(28.6) | 9(60) | 0.036 |
| Rivaroxaban | 6(12) | 5(14.3) | 1(6.7) | 0.447 |
| Dabigatran | 7(14) | 5(14.3) | 2(13.3) | 0.929 |
| Other Drug therapy, n(%) |  |  |  |  |
| Asprin | 9(18) | 8(22.9) | 1(6.7) | 0.172 |
| Clopidogrel | 3(6) | 3(8.6) | 0(0) | 0.242 |
| ACEI/ARB | 17(34) | 16(45.7) | 1(6.7) | 0.008 |
| Alpha-blockers | 3(6) | 0(0) | 3(20) | 0.006 |
| CCB | 9(18) | 8(22.9) | 1(6.7) | 0.172 |
| Diuretics | 14(28) | 7(20) | 7(46.7) | 0.054 |
| MRA | 10(20) | 5(14.3) | 5(33.3) | 0.123 |
| Statins | 15(3) | 14(40.0) | 1(6.7) | 0.018 |
| Antidiabetics | 9(18) | 6(17.1) | 3(20.0) | 0.810 |

Definitions of abbreviations: AF, atrial fibrillation; BSA, body surface area; BMI, body mass index; HCM, hypertrophic cardiomyopathy; IVS, interventricular septum; LVOT, left ventricular outflow tract; ECHO, echocardiography; LA, left atrial; AP, anteroposterior ; LVEDD, left ventricular end-diastolic dimension; LV, left ventricle; HTN, hypertension; CAD, coronary artery disease; DM, diabetes mellitus; CHD, congenital heart disease; VHD, valvular heart disease; PH, pulmonary hypertension; NYHA-FC, New York Heart Association functional class; Non-DHP, Non-dihydropyridine; CCB, calcium channel blocker; ACEI, angiotensin converting enzyme inhibitor; ARB, angiotensin receptor blocker; MRA, mineralocorticoid receptor antagonist.

Values are expressed as n (%), ratio, mean ± SD, or median (interquartile range).

* Comparison between groups of proxysmal and persistent, student t test or Mann-Whiney U test for continuous data, and chi-square test for categorical data, and chi-square test for categorical data.

**Supplemental Table 4. Demographic and clinical characteristics in all patients with SVA underwent ablation**

| **Parameters** | **Overall**  **(n = 34)** | **AF**  **(n = 16)** | **AFL**  **(n = 7)** | **AVRT/AVNRT**  **(n = 13)** |
| --- | --- | --- | --- | --- |
| Male gender, n(%) | 19(55.9) | 5(31.3) | 4(57.1) | 10(76.9) |
| Weight, kg | 68 ± 15.2 | 70 ± 16.4 | 59 ± 13.6 | 67 ± 14.3 |
| Height, cm | 169 ± 8.4 | 167 ± 10.4 | 166 ± 7.3 | 170 ± 6.4 |
| BSA, m^2^ | 1.77 ± 0.21 | 1.78 ± 0.25 | 1.64 ± 0.19 | 1.76 ± 0.19 |
| BMI, kg/m^2^ | 23.7 ± 4.36 | 25.0 ± 4.44 | 21.0 ± 3.77 | 23.0 ± 4.16 |
| Age at admission, y | 52 ± 15.5 | 59 ± 11.7 | 41.6 ± 13.7 | 52 ± 17.8 |
| Age at arrhythmia symptom onset, y | 47 ± 16.9 | 54 ± 13.3 | 40 ± 14.1 | 44 ± 20.6 |
| Age at arrhythmia diagnosis, y | 51 ± 16.1 | 58 ± 12.7 | 40 ± 14.0 | 50 ± 17.8 |
| HCM types |  |  |  |  |
| IVS | 19(55.9) | 11(68.8) | 3(42.9) | 6(46.2) |
| Apex | 2(5.9) | 1(6.3) | 0(0) | 1(7.7) |
| Multi-segment | 13(38.2) | 4(25) | 4(57.1) | 6(46.2) |
| LVOT Obstructive, n(%) | 7(20.6) | 6(37.5) | 0(0) | 1(7.7) |
| History of syncope, n(%) | 2(5.9) | 1(6.3) | 1(14.3) | 0(0) |
| Family history of HCM, n(%) | 2(5.9) | 1(6.3) | 1(14.3) | 0(0) |
| ECHO parameters |  |  |  |  |
| Ejection fraction, % | 61 ± 8.1 | 61 ± 8.9 | 58 ± 3.1 | 64 ± 8.3 |
| LA dimension (AP), mm | 41(13.3) | 43(9.0) | 44(19.0) | 35(12.5) |
| LVEDD, mm | 42 ± 6.4 | 44 ± 6.4 | 39 ± 4.5 | 44 ± 7.3 |
| Max LV thickness, mm | 20(7.3) | 18(4.5) | 22(6.0) | 16(9.0) |
| Mitral regurgitation ≥moderate, n(%) | 0(0) | 0(0) | 0(0) | 0(0) |
| Cormobidities | 14(41.2) | 6(37.5) | 5(71.4) | 5(38.5) |
| HTN, n(%) | 10(29.4) | 6(37.5) | 0(0) | 5(38.5) |
| CAD, n(%) | 5(14.7) | 4(25) | 1(14.3) | 2(15.4) |
| DM, n(%) | 5(14.7) | 3(18.8) | 0(0) | 3(23.1) |
| CHD, n(%) | 2(5.9) | 1(6.3) | 1(14.3) | 0(0) |
| VHD, n(%) | 1(2.9) | 0(0) | 1(14.3) | 0(0) |
| PH, n(%) | 2(5.9) | 1(6.3) | 1(14.3) | 0(0) |
| History of Stroke, n(%) | 4(11.8) | 0(0) | 3(42.9) | 1(7.7) |
| NYHA-FC, n(%) |  |  |  |  |
| I/II | 33(97.1) | 15(93.7) | 7(100) | 13(100) |
| III/IV | 1(2.9) | 1(6.3) | 0(0) | 0(0) |
| Medications |  |  |  |  |
| HCM symptom improving therapy |  |  |  |  |
| Beta-blockers | 21(61.8) | 9(56.3) | 4(57.1) | 8(61.5) |
| Non-DHP-CCB | 5(14.7) | 2(12.5) | 3(42.9) | 0(0) |
| Antiarrhythmia drugs, n(%) |  |  |  |  |
| Amiodarone | 9(26.5) | 5(31.3) | 3(42.9) | 1(7.7) |
| Dronedarone | 1(2.9) | 1(6.3) | 0(0) | 0(0) |
| Propafenone | 3(8.8) | 3(18.8) | 0(0) | 0(0) |
| Sotalol | 4(11.8) | 4(25) | 1(14.3) | 1(7.7) |
| Anticoagulants, n(%) |  |  |  |  |
| Warfarin | 8(23.5) | 5(31.3) | 2(28.6) | 1(7.7) |
| Rivaroxaban | 6(17.6) | 6(37.5) | 0(0) | 1(7.7) |
| Dabigatran | 5(14.7) | 4(25) | 2(28.6) | 0(0) |
| Other Drug therapy, n(%) |  |  |  |  |
| Asprin | 11(32.4) | 0(0) | 1(14.3) | 10(76.9) |
| Clopidogrel | 3(8.8) | 0(0) | 1(14.3) | 2(15.4) |
| ACEI/ARB | 8(23.5) | 5(31.3) | 0(0) | 3(23.1) |
| Alpha-blockers | 1(2.9) | 1(6.3) | 0(0) | 0(0) |
| CCB | 6(17.6) | 4(25) | 0(0) | 1(23.1) |
| Diuretics | 3(8.8) | 1(6.3) | 2(28.6) | 0(0) |
| MRA | 3(8.8) | 2(12.5) | 1(14.3) | 0(0) |
| Statins | 8(23.5) | 4(25) | 1(14.3) | 4(30.8) |
| Antidiabetics | 5(14.7) | 3(18.8) | 0(0) | 3(23.1) |

Definitions of abbreviations: SVA, supraventricular arrhythmia; AF, atrial fibrillation; AFL, atrial flutter; AVRT, atrioventricular reentrant tachycardia; AVNRT, atrioventricular nodal reentrant tachycardia; BSA, body surface area; BMI, body mass index; HCM, hypertrophic cardiomyopathy; IVS, interventricular septum; LVOT, left ventricular outflow tract; ECHO, echocardiography; LA, left atrial; AP, anteroposterior ; LVEDD, left ventricular end-diastolic dimension; LV, left ventricle; HTN, hypertension; CAD, coronary artery disease; DM, diabetes mellitus; CHD, congenital heart disease; VHD, valvular heart disease; PH, pulmonary hypertension; NYHA-FC, New York Heart Association functional class; Non-DHP, Non-dihydropyridine; CCB, calcium channel blocker; ACEI, angiotensin converting enzyme inhibitor; ARB, angiotensin receptor blocker; MRA, mineralocorticoid receptor antagonist.

Values are expressed as n (%), ratio, mean ± SD, or median (interquartile range).

**Supplemental Table 5. Characteristics of patients with AF underwent ablation**

| **Parameters** | **All**  **(n = 16)** | **Recurrence**  **(n = 5)** | **No recurrence**  **(n = 11)** | **P value*** |
| --- | --- | --- | --- | --- |
| Male gender, n(%) | 5 (31.3) | 3 (60.0) | 2 (18.2) | 0.094 |
| Weight, kg | 72 (23.3) | 75 (15.0) | 65 (26.0) | 0.320 |
| Height, cm | 165 (15.8) | 172 (20.5) | 160 (15.0) | 0.145 |
| BSA, m^2^ | 1.81 (0.42) | 1.82 (0.33) | 1.63 (0.40) | 0.267 |
| BMI, kg/m^2^ | 25.0 (5.62) | 27.3 (4.10) | 25.0 (6.23) | 0.510 |
| Age at admission, y | 59 ± 11.8 | 52 ± 12.2 | 61 ± 10.8 | 0.153 |
| Age at arrhythmia symptom onset, y | 54 ± 13.3 | 47 ± 15.4 | 57 ± 11.6 | 0.155 |
| Age at arrhythmia diagnosis, y | 58 ± 12.7 | 53 ± 12.8 | 60 ± 12.5 | 0.304 |
| HCM types |  |  |  | 0.125 |
| IVS | 11 (68.8) | 4 (80) | 7 (63.6) |  |
| Apex | 1 (6.3) | 1 (20) | 0 (0) |  |
| Multi-segment | 4 (25.0) | 0 (0) | 4 (36.4) |  |
| LVOT Obstruction, n(%) | 6 (37.5) | 2 (40) | 4 (36.4) | 0.889 |
| History of syncope, n(%) | 1 (6.3) | 0 (0) | 1 (9.1) | 0.486 |
| Family history of HCM, n(%) | 1 (6.3) | 1 (20) | 0 (0) | 0.126 |
| ECHO parameters |  |  |  |  |
| Ejection fraction, % | 61 (10.3) | 55 (22.3) | 62 (3.0) | 0.661 |
| LA dimension (AP), mm | 43 (9.0) | 44 (10.5) | 43 (12.0) | 0.661 |
| LVEDD, mm | 46 (6.8) | 48 (4.5) | 45 (6.0) | 0.009 |
| Max LV thickness, mm | 18.8 ± 4.2 | 16.2 ± 7.0 | 19.9 ± 4.1 | 0.105 |
| Mitral regurgitation ≥moderate, n(%) | 1 (6.3) | 0 (0) | 1 (9.1) | 0.259 |
| Comorbidities |  |  |  |  |
| HTN, n(%) | 6 (37.5) | 1 (20) | 5 (45.5) | 0.330 |
| CAD, n(%) | 4 (25.0) | 0 (0) | 4 (36.4) | 0.119 |
| DM, n(%) | 3 (18.8) | 0 (0) | 3 (27.3) | 0.195 |
| CHD, n(%) | 1 (6.3) | 0 (0) | 1 (9.1) | 0.486 |
| VHD, n(%) | 0 (0) | 0 (0) | 0 (0) |  |
| PH, n(%) | 1 (6.3) | 1 (20) | 0 (0) | 0.126 |
| History of Stroke, n(%) | 0 (0) | 0 (0) | 0 (0) |  |
| NYHA-FC, n(%) |  |  |  | 0.330 |
| I/II | 15 (92.7) | 5 (100) | 10 (90.9) |  |
| III/IV | 1 (6.3) | 0 (0) | 1 (9.1) |  |
| Antiarrhythmia drugs, n(%) |  |  |  |  |
| Amiodarone | 5 (31.3) | 2 (40) | 3 (27.3) | 0.611 |
| Dronedarone | 1 (6.3) | 0 (0) | 1 (9.1) | 0.486 |
| Propafenone | 3 (18.8) | 1 (20) | 2 (18.2) | 0.931 |
| Sotalol | 4 (25.0) | 0 (0) | 4 (36.4) | 0.119 |

Definitions of abbreviations: TACHY, tachyarrhythmia; BRADY, bradyarrhythmia; BSA, body surface area; BMI, body mass index; HCM, hypertrophic cardiomyopathy; IVS, interventricular septum; LVOT, left ventricular outflow tract; ECHO, echocardiography; LA, left atrial; AP, anteroposterior ; LVEDD, left ventricular end-diastolic dimension; LV, left ventricle; HTN, hypertension; CAD, coronary artery disease; DM, diabetes mellitus; CHD, congenital heart disease; VHD, valvular heart disease; PH, pulmonary hypertension; NYHA-FC, New York Heart Association functional class.

Values are expressed as n (%), ratio, mean ± SD, or median (interquartile range).

* Comparison between patients with and without recurrences, student t test or Mann-Whiney U test for continuous data, and chi-square test for categorical data, and chi-square test for categorical data.
